# Topological Properties of Electrons in Honeycomb Lattice with Detuned Hopping Energy

**DOI:** 10.1038/srep24347

**Published:** 2016-04-14

**Authors:** Long-Hua Wu, Xiao Hu

**Affiliations:** 1 International Center for Materials Nanoarchitectonics (WPI-MANA), National Institute for Materials Science, Tsukuba 305-0044, Japan; 2 Graduate School of Pure and Applied Sciences, University of Tsukuba, Tsukuba 305-8571, Japan

## Abstract

Honeycomb lattice can support electronic states exhibiting Dirac energy dispersion, with graphene as the icon. We propose to derive nontrivial topology by grouping six neighboring sites of honeycomb lattice into hexagons and enhancing the inter-hexagon hopping energies over the intra-hexagon ones. We reveal that this manipulation opens a gap in the energy dispersion and drives the system into a topological state. The nontrivial topology is characterized by the 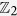 index associated with a pseudo time-reversal symmetry emerging from the *C*_6_ symmetry of the hopping texture, where the angular momentum of orbitals accommodated on the hexagonal “artificial atoms” behaves as the pseudospin. The size of topological gap is proportional to the hopping-energy difference, which can be larger than typical spin-orbit couplings by orders of magnitude and potentially renders topological electronic transports available at high temperatures.

Honeycomb lattice can host electrons with Dirac-like linear dispersion due to its *C*_3_ crystal symmetry^1^, and interests in questing for systems with honeycomb lattice structure flourished since the discovery of graphene produced by the scotch-tape technique^2^,^3^,^4^. The Dirac dispersion and the associated chiral property of electronic wave functions accommodated on honeycomb lattice make it an ideal platform for exploring topological states^5^,^6^ without external magnetic field. It was shown first that a quantum anomalous Hall effect (QAHE) can be realized when complex hopping integral among next-nearest-neighboring sites of honeycomb lattice are taken into account^7^. Later on it was revealed that the intrinsic spin-orbit coupling (SOC) in honeycomb lattice can provide this complex hopping integral, which drives spinful electrons into a topological state with preserved time-reversal (TR) symmetry, known as quantum spin Hall effect (QSHE)^8^,^9^,^10^,^11^. Quite a number of activities have been devoted towards realizing topological states in electron systems on honeycomb lattice, such as QAHE by straining^12^,^13^, twisting^14^,^15^ and decorating graphene^16^, QSHE and QAHE in terms of on-site *p*_*x,y*_ orbitals^17^, and QAHE with spin-polarized edge currents in terms of the antiferromagnetic exchange field and staggered electric potential^18^,^19^. Honeycomb lattice has also been explored to support topological states in photonic crystals^20^,^21^ and cold atoms with on-site *p* orbitals^22^.

In the present work, we explore possible topological properties in honeycomb lattice by introducing a texture in hopping energy between *nearest-neighboring* (NN) sites. We take a hexagonal primitive unit cell and view the honeycomb lattice as a triangle lattice of hexagons [see the dashed red line in Fig. 1(a)]. When the real-valued inter-hexagon hopping *t*_1_ is tuned to be larger than the intra-hexagon one *t*_0_, a topological gap is opened at the Γ point accompanied by a band inversion between orbitals with opposite spatial parities accommodated on hexagons [see Fig. 1(b)]. A pseudo-TR symmetry associated with a pseudospin degree of freedom and Kramers doubling in the emergent orbitals are revealed based on *C*_6_ point group symmetry, which generates the 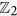 topology. For experimental implementations, we discuss that, along with many other possibilities, the *molecular graphene* realized by placing carbon monoxides (CO) periodically on Cu [111] surface^13^ is a promising platform to realize the present idea, where the hopping texture can be controlled by adding extra CO molecules.

## Results

### Hopping texture and emergent orbitals

**Figure 1:**
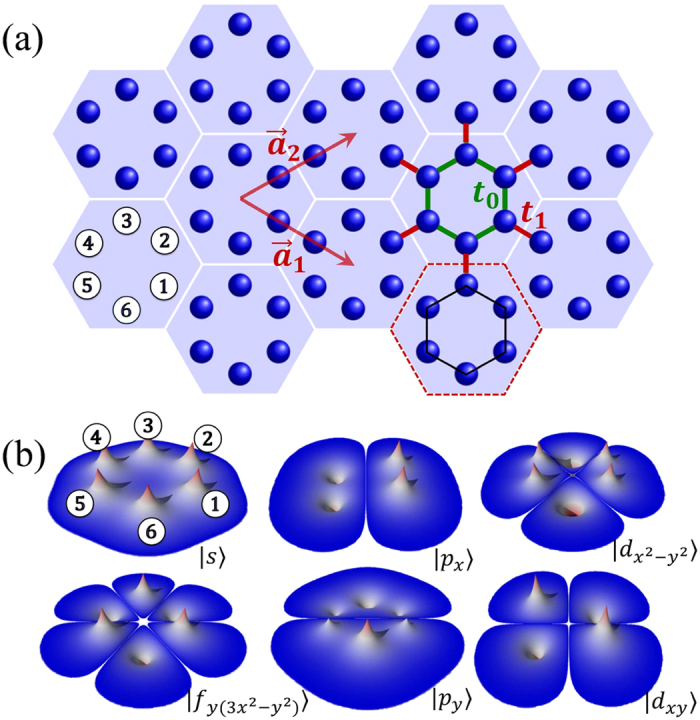
Hopping texture in honeycomb lattice and emergent orbitals. (**a**) Honeycomb lattice with hopping energies between NN sites: *t*_0_ inside hexagons as denoted by the green bonds and *t*_1_ between hexagons by red ones. The red dashed hexagon is the primitive cell of triangular lattice with lattice vectors 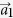, 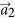 and lattice constant 
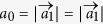
. Numbers 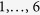 in circle index atomic sites within a hexagon. (**b**) Emergent orbitals in the hexagonal artificial atom.

We start from a spinless tight-binding Hamiltonian on honeycomb lattice^23^


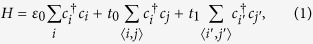


where *c*_*i*_ is the annihilation operator of electron at atomic site *i* with on-site energy *ε*_0_ satisfying the anti-commutation relation, 〈*i*, *j*〉 and 〈*i*′, *j*′〉 run over NN sites inside and between hexagonal unit cells with hopping energies *t*_0_ and *t*_1_ respectively [see Fig. 1(a)]. The orbitals are considered to be the simplest one without any internal structure, such as the *π* electron of graphene. Below we are going to detune the hopping energy *t*_1_ while keeping *t*_0_ constant, and elucidate possible changes in the electronic state. In this case, the pristine honeycomb lattice of individual atomic sites is better to be considered as a triangular lattice of hexagons, with the latter characterized by *C*_6_ symmetry.

Let us start with the Hamiltonian within a single hexagonal unit cell


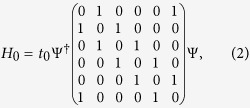


where Ψ = [*c*^1^, *c*^2^, *c*^3^, *c*^4^, *c*^5^, *c*^6^]^*T*^ [see Fig. 1(a)]. The eigen states of Hamiltonian *H*_0_ are given by


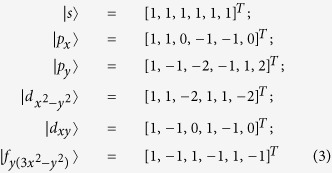


with eigen energies 2*t*_0_, *t*_0_, *t*_0_, −*t*_0_, −*t*_0_ and −2*t*_0_ respectively, up to normalization factors. As shown in Fig. 1(b), the *emergent* orbitals accommodated on the hexagonal “artificial atom” take the shapes similar to the conventional *s*, *p*, *d* and *f* atomic orbitals.

It is easy to check that the wave functions


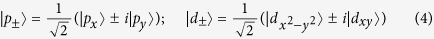


are related each other by the operator 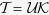: 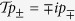 and 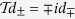 with 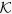 the complex conjugate operator and 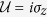, where *σ*_*z*_ is the Pauli matrix. Therefore, the operator 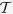 can be taken as a pseudo-TR operator, and the orbital angular momentum plays the role of a pseudospin. The relation 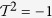 yields the Kramers doubling, a property originating from the *C*_6_ symmetry. It is noticed that the high-energy states |*s*〉 and 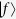 are singlets, and thus the pseudospin and pseudo-TR symmetry are valid only for low-energy physics, which however is sufficient for realizing nontrivial topological properties in the present system (see also ref. 21).

**Figure 2:**
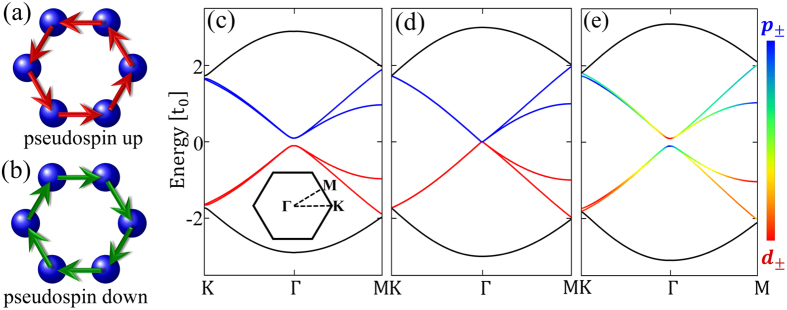
Band inversion and topological phase transition. (**a**,**b**) Current densities in the pseudospin-up channel (*p*_+_ or *d*_+_) and pseudospin-down channel (*p*_−_ or *d*_−_) respectively. Band dispersions for the system given in Fig. 1: (**c**) *t*_1_ = 0.9*t*_0_ (Inset: Brillouin zone of the triangular lattice), (**d**) *t*_1_ = *t*_0_ and (**e**) *t*_1_ = 1.1*t*_0_. Blue and red are for |*p*_±_〉 and |*d*_±_〉 orbitals respectively, and rainbow for hybridization between them. The on-site energy is taken *ε*_0_ = 0.

Distinguished from the intrinsic spin, the pseudospin is directly related to the chiral current density on the hexagon. For a lattice model, the current density between two sites is given by 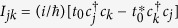. The current distributions evaluated using wave functions in Eq. (4) for the pseudospin-up and -down states are shown in Fig. 2(a,b) with anticlockwisely and clockwisely circulating currents. By considering the hexagonal artificial atoms composed by six sites in honeycomb lattice, one harvests states with angular momenta merely from simple orbitals, such as *π* electrons in graphene. The pseudo-TR symmetry is supported by the *C*_6_ crystal symmetry, sharing the same underlying physics with the topological crystalline insulator^24^. However, for crystal-symmetry-protected topological insulators addressed so far, strong SOCs are required to achieve band inversions^25^,^26^,^27^, which is different from the present approach as revealed below.

### Topological phase transition

We calculate the energy dispersion of Eq. (1) for several typical values of *t*_1_ (hereafter the on-site energy is put as *ε*_0_ = 0 without losing generality). As shown in Fig. 2, there are two two-fold degeneracies at the Γ point corresponding to the two two-dimensional (2D) representations of *C*_6_ point group. Projecting the wave functions for *t*_1_ = 0.9*t*_0_ onto the orbitals given in Fig. 1(b), it is found that the topmost two valance bands show the character of *d* orbitals whereas the lowest two conduction bands behave like *p* orbitals [see Fig. 2(c)], with the order in energy same as those listed in Eq. (3). For *t*_1_ = *t*_0_, the *d* and *p* bands become degenerate at the Γ point and double Dirac cones appear [see Fig. 2(d)], which are equivalent to the ones at *K* and *K*′ points in the unfolded Brillouin zone of honeycomb lattice with the rhombic unit cell of two sites. When *t*_1_ increases further from *t*_0_, a band gap reopens at the Γ point. As shown in Fig. 2(e) for *t*_1_ = 1.1*t*_0_, the valence (conduction) bands are now occupied by *p* (*d*) orbitals around the Γ point, opposite to the order away from the Γ point and to that before gap closing. Therefore, a band inversion between *p* and *d* orbitals takes place at the Γ point when the inter-hexagon hopping energy is increased across the topological transition point *t*_1_ = *t*_0_, namely the pristine honeycomb lattice.

We can characterize the topological property of the gap-opening transition shown in Fig. 2 by a low-energy effective Hamiltonian around the Γ point. Since the bands near the Fermi level are predominated by *p* and *d* orbitals, it is sufficient to downfold the six-dimensional Hamiltonian *H*(**k**) associated with the tight-binding model (1) into the four-dimensional subspace [*p*_+_, *d*_+_, *p*_−_, *d*_−_]. The second term in Eq. (1) is then simply given by


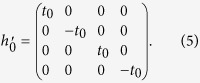


Contributions from the third term in Eq. (1) to the effective Hamiltonian can be evaluated in the following way^28^. First, we list the inter-hexagon hoppings in terms of 6 × 6 matrices 
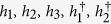
 and 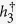 with


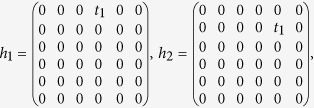



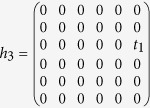


on the basis of [*c*^1^, *c*^2^, *c*^3^, *c*^4^, *c*^5^, *c*^6^]. Following the standard procedures^28^, they can be projected to the subspace spanned by [*p*_+_, *d*_+_, *p*_−_, *d*_−_]


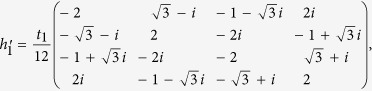



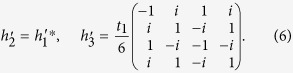


With Fourier transformations of matrices in Eqs. (5) and (6), one obtains the effective low-energy Hamiltonian *H*′(**k**) on the basis [*p*_+_, *d*_+_, *p*_−_, *d*_−_] in the momentum space. Expanding *H*′(**k**) around the Γ point up to the lowest-orders of **k**, one arrives at


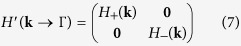


with


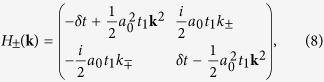


where 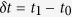, **k** = (*k*_*x*_, *k*_*y*_), *k*_±_ = *k*_*x*_ ± *ik*_*y*_, **0** is a 2 × 2 zero matrix, and *a*_0_ is the lattice constant of the triangular lattice. For *δt* = 0, the Hamiltonians *H*_+_(**k**) and *H*_−_(**k**) in Eq. (8) are the same as the well-known one for honeycomb lattice around the *K* and *K*′ points^29^, where the quadratic terms of momentum in the diagonal parts can be neglected.

For *δt* > 0, however, the quadratic terms are crucially important since they induce a band inversion^30^, resulting in the orbital hybridization in the band structures shown in Fig. 2(e). Associated with a skyrmion in the momentum space for the orbital distributions in the individual pseudospin channels, a topological state appears characterized by the 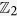 topological invariant^10^,^11^,^21^,^31^. It is clear that for *δt* < 0 there is no band inversion taking place and thus the band gap is trivial as shown in Fig. 2(c).

The pseudo-TR symmetry satisfying 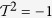 is preserved at the Γ point. Going away from the Γ point, the *C*_6_ symmetry is gradually broken, so does the pseudo-TR symmetry, and the two pseudospin channels start to mix with each other. However, this mixing is weak around the Γ point where the topological property of the system is determined. This can be seen directly from the band structure shown in Fig. 2(e) [as well as in Fig. 2(c,d)], where a gap between *p*_+_ and *p*_−_ (*d*_+_ and *d*_−_) is hardly observed around the Γ point. Analytically, the matrix elements between the two pseudospin channels in the Hamiltonian (7) are quadratic in momentum, which can be neglected as high-order corrections when the topological property is addressed.

It is worthy noticing that, comparing with the Kane-Mele model for the honeycomb lattice^10^,^11^, the mass term *δt*(>0) in Eq. (8) can be considered as an effective SOC associated with the pseudospin, namely *λ*_eSOC_ = *δt*. For *δt* = 0.1*t*_0_, a moderate texture in hopping energies, the effective SOC is approximately 3000 times larger than the *real* SOC in magnitude in graphene where *λ*_SOC_ ≃ 0.1 meV and *t*_0_ = 2.7 eV. The huge effective SOC is due to its pure electronic character as compared with the intrinsic SOC originated from the relativistic effect. This is one of the fantastic aspects of the present approach, which renders a topological gap corresponding to temperature of thousands of Kelvin.

### Topological edge states and associated conductances

**Figure 3:**
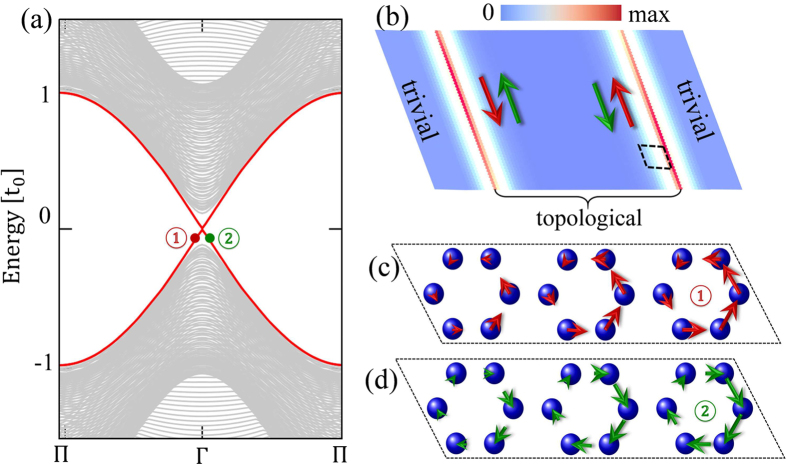
Topological edge states. (**a**) Band dispersion of a ribbon system of 36 hexagons with *t*_1_ = 1.1*t*_0_ cladded from both sides by 10 hexagons with *t*_1_ = 0.9*t*_0_. (**b**) Real-space distribution of the in-gap states associated with the red solid dispersion curves in (**a**). (**c**,**d**) Real-space distributions of current densities in pseudospin-up and -down channels at the momenta indicated by the red and green dots 1 and 2 in (**a**) within the rhombic area sketched by dashed line in (**b**); the excess currents in pseudospin-up and -down channels are indicated by red and green arrows in (**b**).

We consider a ribbon of hexagonal unit cells of *t*_1_ = 1.1*t*_0_ with its two edges cladded by hexagonal unit cells of *t*_1_ = 0.9*t*_0_. As can be seen in Fig. 3(a), additional states appear in the bulk gap as indicated by the red solid curves carrying double degeneracy. Plotting the spatial distribution of the corresponding wave functions, we find that the in-gap states are localized at the two interfaces between topological and trivial regions [see Fig. 3(b)]. As displayed in Fig. 3(c,d), there is an excess upward (downward) edge current in the pseudospin-up (-down) channel associated with the state indicated by the red (green) dot in Fig. 3(a).

**Figure 4:**
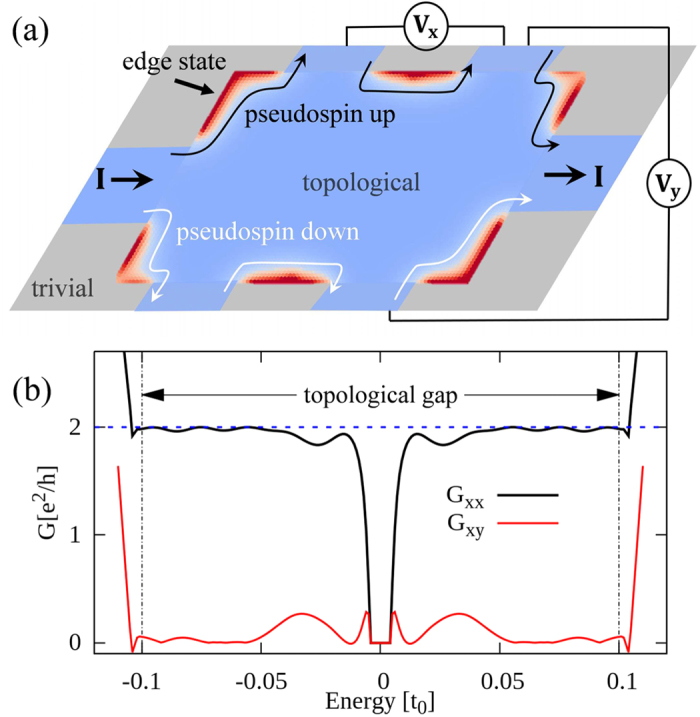
Conductances of the topological phase. (**a**) Schematic configuration of a six-terminal Hall bar where a topological sample (light blue region) with *t*_1_ = 1.1*t*_0_ is embedded in a trivial environment (gray region) with *t*_1_ = 0.9*t*_0_. The size of topological scattering region is 240*a*_0_ × 120*a*_0_, and the width of each semi-infinite lead is 40*a*_0_. The injected current flows along the edges of topological sample as indicated by the red parts between electrodes. (**b**) Longitudinal and Hall conductances of the Hall bar as a function of energy of incident electrons. The on-site energy is taken *ε*_0_ = 0. A rhombic topological sample is taken for ease of calculation.

At the interface between topological and trivial regimes, the crystal symmetry is reduced from *C*_6_ to *C*_2_, which breaks the pseudo-TR symmetry in contrast to the real TR symmetry. As the result, a mini gap of ~0.01*t*_0_ [unnoticeable in the scale of Fig. 3(a)] opens in the edge states at the Γ point due to the coupling between two pseudospin channels. In order to quantitatively check possible backscatterings caused by this mini gap, we perform calculations on the longitudinal and Hall conductances based on a Hall bar system as sketched in Fig. 4(a). It is clear that the current *I* injected from the left electrode divides itself into two branches according to the pseudospin states, namely pseudospin-up (-down) electrons can flow only in the upper (lower) edge of the Hall bar. By matching wave functions at the interfaces between the six semi-infinite electrodes and the topological scattering region^32^,^33^, one can evaluate the transmissions of plane waves scattered among all the six leads, and then the longitudinal and Hall conductances, 
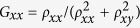
 and 
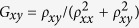
 respectively, by the Landauer-Büttiker formalism^34^, where *ρ*_*xx*_ and *ρ*_*xy*_ are the longitudinal and transverse resistances, respectively. Similar to the case of QSHE with magnetic impurities^35^, the values of conductivity in the present system deviate from the quantized ones when the Fermi level falls in the mini gap of ~0.01*t*_0_ as shown in Fig. 4(b). It is noticed, however, that both *G*_*xx*_ and *G*_*xy*_ heal quickly after several periods of oscillations that come from interferences between the two pseudospin channels. It is emphasized that almost perfectly quantized conductances *G*_*xx*_ = 2*e*^2^/*h* and *G*_*xy*_ = 0^30^,^36^ are achieved for the Fermi level beyond 0.04*t*_0_ up to the bulk gap edge at 0.1*t*_0_, where the edge states with almost perfect linear dispersions hardly feel the existence of the mini gap and essentially no appreciable backscattering exists. On the other hand, if the inter-hexagon hopping energy is put far away from the intra-hexagon one in topological and/or trivial regimes, edge states may hardly be noticed^37^.

**Figure 5:**
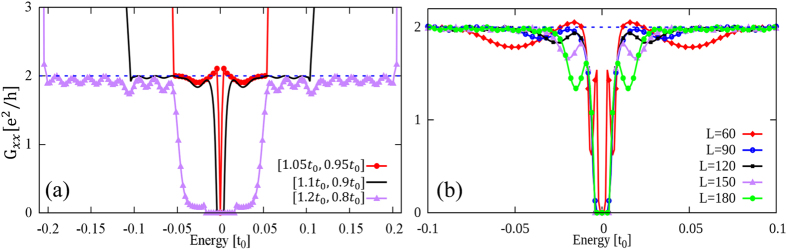
Hopping-energy and sample-size dependence of longitudinal conductances. Longitudinal conductance *G*_*xx*_ of the topological sample given in Fig. 4(a) as a function of the energy of injected electrons: (**a**) for several typical values of inter-hexagon hopping integrals [1.05*t*_0_, 0.95*t*_0_], [1.1*t*_0_, 0.9*t*_0_] and [1.2*t*_0_, 0.8*t*_0_], where the first (second) inside bracket is for the topological (trivial) region; (**b**) with several typical system sizes 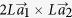, where the width of the electrodes is fixed at 40*a*_0_ and the inter-hexagon hopping integral is fixed at *t*_1_ = 1.1*t*_0_ and *t*_1_ = 0.9*t*_0_ for the topological and trivial regions respectively.

Now we investigate the hopping-energy dependence of the longitudinal conductance. The size of scattering region is same as in Fig. 4(a) and fixed for all cases. As displayed in Fig. 5(a), *G*_*xx*_ saturates to the quantized value 2*e*^2^/*h* as expected for a 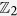 topological state for all the cases with *t*_1_ = 1.05*t*_0_, 1.1*t*_0_ and 1.2*t*_0_ in the topological region (whereas 0.95*t*_0_, 0.9*t*_0_ and 0.8*t*_0_ in the trivial region correspondingly) when the Fermi level is set away from the mini gaps, accompanied by oscillations due to interferences between the two pseudospin channels.

We then check the sample-size dependence of the longitudinal conductance. We fix inter-hexagon hopping integrals at 1.1*t*_0_ and 0.9*t*_0_ in the topological and trivial regions respectively. As displayed in Fig. 5(b), *G*_*xx*_ saturates in all cases to the quantized value 2*e*^2^/*h* when the Fermi level is shifted away from the mini gap. The topological edge transports remain unchanged when the size of the topological region becomes large.

### Real spin and QSHE

In addition to the pseudospin, the *real* spin degree of freedom also contributes to transport properties. In absence of the real SOC, the results presented in Fig. 4 remain exactly the same, with an additional double degeneracy due to the two spin channels and thus *G*_*xx*_ = 4*e*^2^/*h*.

**Figure 6:**
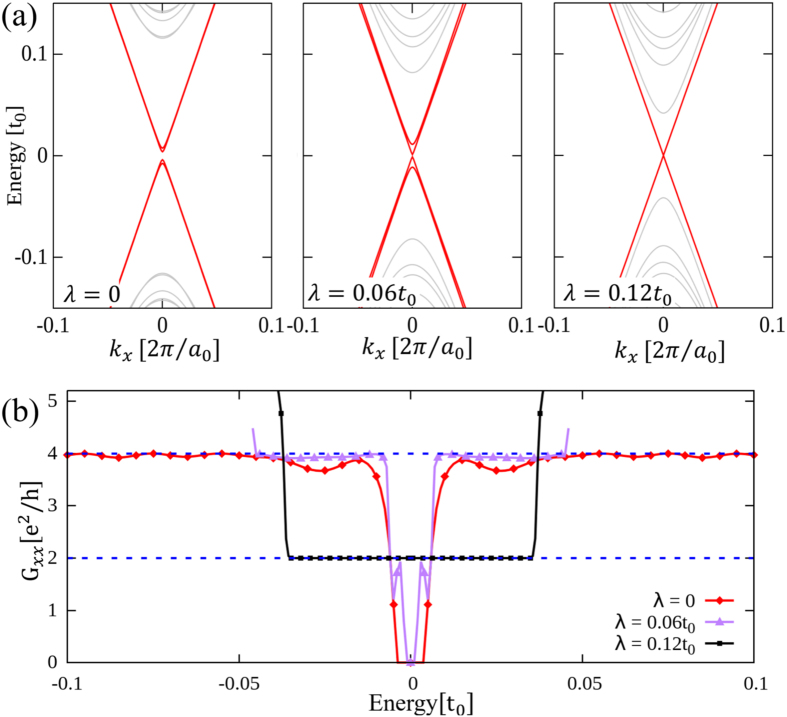
Edge states and conductances of the topological phase in the presence of SOC. (**a**) Dispersion relations and (**b**) longitudinal conductances of the topological system same as that given in Fig. 4(a) except that finite SOC is included.

An intrinsic SOC is induced when next-nearest-neighbor hoppings in honeycomb lattice are taken into account^10^,^11^. The low-energy Hamiltonian around the Γ point in Eq. (7) is then modified to


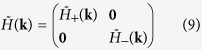


with


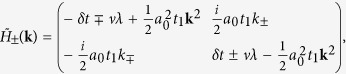


where *ν* = 1 and −1 stand for spin-up and -down states respectively. Therefore, in the spin-up channel SOC enhances (suppresses) the topological gap in the pseudospin-up (-down) channel presuming *λ* > 0 [see the left and central panels of Fig. 6(a)]. As far as *λ* < *δt*, the system remains the 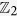 topological state associated with the pseudospin, where electrons with up pseudospin and down pseudospin counter propagate at edges, both carrying on up and down spins. The longitudinal conductance *G*_*xx*_ saturates to 4*e*^2^/*h* as displayed in Fig. 6(b).

When SOC is increased to *λ* = *δt*, the pseudospin-down (-up) channel with up (down) spin is driven into a semi-metallic state with zero band gap and the Dirac dispersion appears at the Γ point. When SOC goes beyond *δt*, this Dirac dispersion opens a gap accompanied by a topological phase transition. The system now takes a QSHE state where at edges electrons with up spin and pseudospin propagate oppositely to electrons with down spin and pseudospin. Evaluating the longitudinal conductance, one finds that *G*_*xx*_ is quantized exactly to 2*e*^2^/*h* [see Fig. 6(b)], and as shown in the right panel of Fig. 6(a) there is no mini gap in the edge states, as protected by *real* TR symmetry^10^,^11^.

## Discussions

A tight-binding model for spinless fermions on honeycomb lattice was considered in a previous work with a Kekulé pattern in nearest-neighbor hopping integrals^23^. It was revealed that fractional charges can be achieved when the mass gap contains a vortex, which requires the hopping integral depending on position in a special way. In contrast, in the present model Hamiltonian (1) there are only two values of hopping integral, and when the inter-hexagon one is larger than the intra-hexagon one a 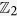 topological state characterized by emergent pseudospin degree of freedom appears.

**Figure 7:**
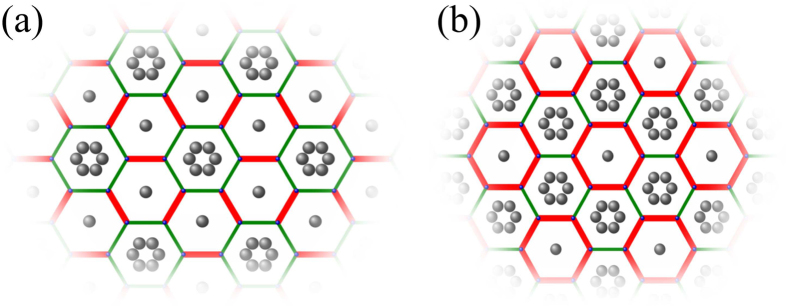
Schematics for hopping textures with *C*_6_ symmetry. Molecular graphene realized by decorating the Cu [111] surface with a triangular lattice of CO molecules: (**a**) *t*_1_ > *t*_0_ generating topological state, (**b**) *t*_1_ < *t*_0_ for trivial state. Gray balls are CO molecules decorated by STM techniques, and red thick bonds are shorter than green thin ones which generates the hopping textures.

We then discuss possible experimental realization of our theoretical proposal. Much effort has been devoted towards realizing the Dirac-like energy dispersion in artificial honeycomb lattices^38^, ranging from optical lattices^39^,^40^ to 2D electron gases modulated by periodic potentials^13^,^41^,^42^ and In_2_Te_2_/graphene bilayers^43^. All these systems provide promising platforms for realizing topological properties by detuning effective hopping energy among NN sites either by modulating muffin-tin potentials or bond lengthes periodically. To be specific, here we focus on how to achieve a topological state on the Cu [111] surface modulated by triangular gates of carbon monoxide (CO) molecules^13^. When extra CO molecules are placed at specific positions over the pristine molecular graphene, the bonds of the hexagons surrounding them are elongated since the CO clusters enhance local repulsive potentials and push electrons away from them, which reduces the corresponding electron hopping energies^42^. It is extremely interesting from the present point of view that hopping textures with *C*_6_ symmetry have already been achieved in experiments^13^. We propose to place extra CO atoms in the pattern displayed in Fig. 7(a), where the intra-hexagon hopping energy *t*_0_ (green thin bonds) surrounding the CO clusters is reduced and the inter-hexagon hopping energy *t*_1_ (red thick bonds) is enhanced relatively. According to the above discussions, the system displayed in Fig. 7(a) with *t*_1_ > *t*_0_ should take a topological state. The hopping texture in Fig. 7(b), dual to that shown in Fig. 7(a), was realized in experiments^13^, where the system takes a topologically trivial state because *t*_1_ < *t*_0_ (see also ref. 44).

The underlying idea of the present scheme for achieving the 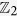 topological state is to create artificial orbitals carrying on opposite orbital angular momenta and parities with respect to spatial-inversion symmetry, and to induce a band inversion between them by introducing the hopping texture with *C*_6_ symmetry on honeycomb lattice. In the sense that it does not require SOC, the present state may be understood as a quantum *orbital* Hall effect. The topological properties can also be extended to photonic crystals^21^, cold atoms, physical systems of exciton, polariton, surface plasmon, and phonon.

In conclusion, we propose to derive topological properties by modulating electron hopping energy between nearest-neighbor sites of honeycomb lattice. Because of the hopping texture with *C*_6_ symmetry, atomic-like orbitals emerge, which carry a pseudospin degree of freedom characterizing a pseudo time-reversal symmetry and rendering Kramers pairs. We reveal that the effective spin-orbit coupling associated with the pseudospin degree of freedom can be larger than the intrinsic one by orders of magnitude because of the pure electronic origin. The present work offers a new possibility for achieving topological properties and related novel quantum properties and functionalities at high temperatures.

## Methods

Energy dispersion relations are obtained by direct diagonalizations of Hamiltonian. Calculations of longitudinal and Hall conductances are performed by using open software Kwant^33^.

## Additional Information

**How to cite this article**: Wu, L.-H. and Hu, X. Topological Properties of Electrons in Honeycomb Lattice with Detuned Hopping Energy. *Sci. Rep.* **6**, 24347; doi: 10.1038/srep24347 (2016).

## References

[b1] Wallace P. R. The Band Theory of Graphite. Phys. Rev. 71, 622–634 (1947).

[b2] Novoselov K. S. *et al.* Electric Field Effect in Atomically Thin Carbon Films. Science 306, 666–669 (2004). 15499015 10.1126/science.1102896

[b3] Katsnelson M. I. Graphene Carbon in Two dimensions. (Cambridge University Press, 2012).

[b4] Geim A. K. Graphene: Status and Prospects. Science 324, 1530–1534 (2009). 19541989 10.1126/science.1158877

[b5] Klitzing K. v., Dorda G. & Pepper M. New Method for High-Accuracy Determination of the Fine-Structure Constant Based on Quantized Hall Resistance. Phys. Rev. Lett. 45, 494 (1980).

[b6] Thouless D. J., Kohmoto M., Nightingale M. P. & den Nijs M. Quantized Hall Conductance in a Two-Dimensional Periodic Potential. Phys. Rev. Lett. 49, 405–408 (1982).

[b7] Haldane F. D. M. Model for a Quantum Hall Effect without Landau Levels: Condensed-Matter Realization of the “Parity Anomaly”. Phys. Rev. Lett. 61, 2015–2018 (1988). 10038961 10.1103/PhysRevLett.61.2015

[b8] Hasan M. Z. & Kane C. L. *Colloquium*: Topological insulators. Rev. Mod. Phys. 82, 3045–3067 (2010).

[b9] Qi X.-L. & Zhang S.-C. Topological insulators and superconductors. Rev. Mod. Phys. 83, 1057–1110 (2011).

[b10] Kane C. L. & Mele E. J. Quantum Spin Hall Effect in Graphene. Phys. Rev. Lett. 95, 226801 (2005). 16384250 10.1103/PhysRevLett.95.226801

[b11] Kane C. L. & Mele E. J. *Z*_2_ Topological Order and the Quantum Spin Hall Effect. Phys. Rev. Lett. 95, 146802 (2005). 16241681 10.1103/PhysRevLett.95.146802

[b12] Guinea F., Katsnelson M. I. & Geim A. K. Energy gaps and a zero-field quantum Hall effect in graphene by strain engineering. Nature Phys. 6, 30–33 (2010).

[b13] Gomes K. K., Mar W., Ko W., Guinea F. & Manoharan H. C. Designer Dirac fermions and topological phases in molecular graphene. Nature 483, 306–310 (2012). 22422264 10.1038/nature10941

[b14] Hunt B. *et al.* Massive Dirac Fermions and Hofstadter Butterfly in a van der Waals Heterostructure. Science 340, 1427–1430 (2013). 23686343 10.1126/science.1237240

[b15] Yan W. *et al.* Strain and curvature induced evolution of electronic band structures in twisted graphene bilayer. Nat. Commun. 4, 2159 (2013). 23851673 10.1038/ncomms3159

[b16] Qiao Z. *et al.* Quantum anomalous Hall effect in graphene from Rashba and exchange effects. Phys. Rev. B 82, 161414 (2010).

[b17] Zhang G.-F., Li Y. & Wu C. Honeycomb lattice with multiorbital structure: Topological and quantum anomalous Hall insulators with large gaps. Phys. Rev. B 90, 075114 (2014).

[b18] Liang Q.-F., Wu L.-H. & Hu X. Electrically tunable topological state in [111] perovskite materials with an antiferromagnetic exchange field. New J. Phys. 15, 063031 (2013).

[b19] Ezawa M. Spin valleytronics in silicene: Quantum spin Hallquantum anomalous Hall insulators and single-valley semimetals. Phys. Rev. B 87, 155415 (2013).

[b20] Haldane F. D. M. & Raghu S. Possible Realization of Directional Optical Waveguides in Photonic Crystals with Broken Time-Reversal Symmetry. Phys. Rev. Lett. 100, 013904 (2008). 18232766 10.1103/PhysRevLett.100.013904

[b21] Wu L.-H. & Hu X. Scheme for Achieving a Topological Photonic Crystal by Using Dielectric Material. Phys. Rev. Lett. 114, 223901 (2015). 26196622 10.1103/PhysRevLett.114.223901

[b22] Wu C. Orbital Analogue of the Quantum Anomalous Hall Effect in *p*-Band Systems. Phys. Rev. Lett. 101, 186807 (2008). 18999852 10.1103/PhysRevLett.101.186807

[b23] Hou C. Y., Chamon C. & Mudry C. Electron Fractionalization in Two-Dimensional Graphenelike Structures. Phys. Rev. Lett. 98, 186809 (2007). 17501599 10.1103/PhysRevLett.98.186809

[b24] Fu L. Topological Crystalline Insulators. Phys. Rev. Lett. 106, 106802 (2011). 21469822 10.1103/PhysRevLett.106.106802

[b25] Hsieh T. H. *et al.* Topological crystalline insulators in the SnTe material class. Nat. Commun. 3, 982 (2012). 22864575 10.1038/ncomms1969

[b26] Dziawa P. *et al.* Topological crystalline insulator states in Pb_1−*x*_Sn_*x*_Se. Nature Mater. 11, 1023 (2012). 23023551 10.1038/nmat3449

[b27] Xu S. Y. *et al.* Observation of a topological crystalline insulator phase and topological phase transition in Pb_1−*x*_Sn_*x*_Te. Nat. Commun. 3, 1192 (2012). 23149737 10.1038/ncomms2191

[b28] Sakurai J. J. Modern Quantum Mechanics. (Addison Wesley, 1985).

[b29] Neto A. H. C., Guinea F., Peres N. M. R., Novoselov K. S. & Geim A. K. The electronic properties of graphene. Rev. Mod. Phys. 81, 109 (2009).

[b30] Bernevig B. A., Hughes T. L. & Zhang S.-C. Science Quantum Spin Hall Effect and Topological Phase Transition in HgTe Quantum Wells. Science 314, 1757–1761 (2006). 17170299 10.1126/science.1133734

[b31] Fu L. & Kane C. L. Topological insulators with inversion symmetry. Phys. Rev. B 76, 045302 (2007).

[b32] Ando T. Quantum point contacts in magnetic fields. Phys. Rev. B 44, 8017 (1991). 10.1103/physrevb.44.8017 9998733

[b33] Groth C. W., Wimmer M., Akhmerov A. R. & Waintal X. Kwant: a software package for quantum transport. New J. Phys. 16, 063065 (2014).

[b34] Imry Y. & Landauer R. Conductance viewed as transmission. Rev. Mod. Phys. 71, S306–S312 (1999).

[b35] Tkachov G. & Hankiewicz E. M. Ballistic Quantum Spin Hall State and Enhanced Edge Backscattering in Strong Magnetic Fields. Phys. Rev. Lett. 104, 166803 (2010). 20482073 10.1103/PhysRevLett.104.166803

[b36] König M. *et al.* Quantum Spin Hall Insulator State in HgTe Quantum Wells. Science 318, 766–770 (2007). 17885096 10.1126/science.1148047

[b37] Wu W., Rachel S., Liu W. M. & Hur K. L. Quantum spin Hall insulators with interactions and lattice anisotropy. Phys. Rev. B 85, 205102 (2012).

[b38] Polini M., Guinea F., Lewenstein M., Manoharan H. C. & Pellegrini V. Artificial honeycomb lattices for electrons, atoms and photons. Nature Nanotech. 8, 625 (2013). 10.1038/nnano.2013.161 24002076

[b39] Wunsch B., Guinea F. & Sols F. Dirac-point engineering and topological phase transitions in honeycomb optical lattices. New J. Phys. 10, 103027 (2008).

[b40] Tarruell L., Greif D., Uehlinger T., Jotzu G. & Esslinger T. Creating, moving and merging Dirac points with a Fermi gas in a tunable honeycomb lattice. Nature 483, 302–305 (2012). 22422263 10.1038/nature10871

[b41] Gibertini M., Singha A., Pellegrini V. & Polini M. Engineering artificial graphene in a two-dimensional electron gas. Phys. Rev. B, 79, 241406 (2009).

[b42] Park C.-H. & Louie S. G. Making Massless Dirac Fermions from a Patterned Two-Dimensional Electron Gas. Nano Lett. 9, 1793–1797 (2009). 19338276 10.1021/nl803706c

[b43] Giovannetti G., Capone M., Brink J. v. D. & Ortix C. Kekulé textures, pseudospin-one Dirac cones, and quadratic band crossings in a graphene-hexagonal indium chalcogenide bilayer. Phys. Rev. B 91, 121417(R) (2015).

[b44] Park J. S. & Choi H. J. Band-gap opening in graphene: A reverse-engineering approach. Phys. Rev. B 92, 045402 (2015).

